# Intervention Adherence is Related to Participant Retention: Implications for Research

**DOI:** 10.2196/jmir.2948

**Published:** 2014-05-21

**Authors:** John Alastair Cunningham

**Affiliations:** ^1^Centre for Mental Health ResearchThe Australian National UniversityCanberraAustralia; ^2^Centre for Addiction and Mental HealthToronto, ONCanada

**Keywords:** research methods, randomized controlled trials, alcohol, Internet, brief intervention

Two challenging issues in Internet intervention research, as well as in other behavioral intervention trials, are ensuring that participants receive the intervention (adherence) and that their outcomes are captured at follow-up (retention) [1]. The interesting analysis presented by Murray et al [2] demonstrated that, at least in their study sample, the participant adherence and retention were positively related.

One issue to consider is whether this finding can be replicated in other study samples. It is possible that research involving, for example, different recruitment methods or with higher (or lower) retention rates, might not display this same positive relationship. To that purpose, results were examined from an Internet intervention trial that employed a proactive telephone recruitment method and obtained complete follow-up data for 86% of participants [3-5]. As with the Murray et al study [2], adherence (measured by the number of intervention participants logging onto a brief alcohol intervention, where N=92; 57 participants logged onto the intervention and 35 participants did not log on) and retention were strongly positively related (retention at 3-months: logged onto intervention=100%, did not log on=80%, *P*<.001; retention at 6-months: logged onto intervention=100%; did not log on=80%, *P*<.001; retention at 12-months: logged onto intervention=96%; did not log on=74.3%, *P*=.002; Fisher’s Exact Tests).

Given that the positive relationship between adherence and retention can be replicated, what are the implications of this finding? From one perspective, the fact that these two key issues are related could underline the increased importance of obtaining a good retention rate. This is because the positive relationship of adherence to retention implies that a confound in the interpretation of the results is more likely as loss to follow-up (or reduced adherence to the intervention) increases. Alternatively, it could be argued that this positive relationship might reduce the importance of obtaining a good retention rate. This is because traditional intent-to-treat analysis assumes that participants who are lost to follow-up do not make any change in their behavior from baseline to follow-up (and are included as imputed values in the analysis based on this assumption). If it is then assumed that only those participants who accessed the intervention will actually make a change in their behavior, then the fact that participants who adhere to the intervention are more likely to follow-up can only increase the likelihood that participants who are lost to follow-up are less likely to have made a change in their behavior (thus validating the intent-to-treat analysis assumption). Determining which of these implications is correct is important, particularly in a field where low retention rates are an unfortunate reality in many research trials [1].
